# Evaluation of salivary stress markers and inflammatory cytokine levels in peri-implantitis patients

**DOI:** 10.1007/s00784-024-05692-5

**Published:** 2024-05-01

**Authors:** Fatma Soysal, Berrin Unsal, Sila Cagri Isler, Gulcin Akca, Batuhan Bakirarar, Mustafa Ozcan

**Affiliations:** 1 Department of Periodontology, Faculty of Dentistry, Ankara Medipol University, Ankara, Turkey; 2 Department of Periodontology, Faculty of Dentistry, Gazi University, Ankara, Turkey; 3 Department of Periodontology, School of Dental Medicine, University of Bern, Bern, Switzerland; 4 Department of Medical Microbiology, Faculty of Dentistry, Gazi University, Ankara, Turkey; 5 Department of Biostatistics, Faculty of Medicine, Ankara University, Ankara, Turkey; 6 Department of Periodontology, Faculty of Dentistry, Cukurova University, Adana, Turkey

**Keywords:** Peri-implantitis, Psychological stress, Saliva, Biomarkers

## Abstract

**Background and objective:**

Psychological stress has been identified in some observational studies as a potential factor that may modify and affect periodontal diseases, but there are no similar data for peri-implantitis. The aim of this study was to determine the relationship between interleukin (IL)-1β, IL-6, IL-10, interferon (IFN)α inflammatory cytokines and the psychological stress-related markers, glucocorticoid receptor-α (GRα), and salivary α-amylase (sAA) gene expression levels in saliva samples obtained from healthy implants and peri-implantitis patients.

**Materials and methods:**

The study included a total of 50 systemically healthy subjects. Peri-implant clinical parameters were recorded and psychological stress level was evaluated with the hospital anxiety and depression scale (HAD) and state-trait anxiety inventory (STAI) questionnaire forms. Following the evaluations, the patients were divided into 4 groups according their stress and clinical status (Ia, Ib, IIa, IIb). IL-1β, IL-6, IL-10, IFNα, GRα, sAA gene expression levels in the saliva samples were quantified by quantitative polymerase chain reaction (qPCR).

**Results:**

In the group of peri-implantitis who had a high score in stress level assessment scales, significantly higher IL-1β, IL-6, sAA expression levels were observed (*p* < 0.001). The IL-10 gene expression levels were lower in the groups with a high score in the stress level assessment scales (*p* < 0.001). GRα gene was expressed at lower levels in the group of peri-implantitis who had a high score in stress level assessment scales but the difference was not statistically significant (*p* = 0.065).

**Conclusion:**

The study findings suggest that psychological stress may increase the inflammation associated with peri-implantitis by affecting cytokine expression levels.

**Clinical relevance:**

To prevent peri-implantitis or reduce its prevalence, it could be beneficial to evaluate stress levels and identify individuals experiencing stress.

## Introduction

An imbalance among bacterial load and host response results in peri-implant diseases [1]. Peri-implant diseases may affect peri-implant mucosa alone (peri-implant mucositis) or both peri-implant mucosa and supporting bone (peri-implantitis) [2]. Peri-implantitis and peri-implant mucositis are differentiated by the match around the teeth; periodontitis and gingivitis. Both periodontitis and peri-implantitis have common features in terms of clinical features, etiology, pathogenesis, therapy and risk factors [3]. Studies have shown that the major risk factors for periodontal disease, such as poor oral hygiene and tobacco consumption, also represent risk indicators for peri-implantitis. Diabetes, alcohol consumption, and genetic traits have also been suggested as risk factors for peri-implantitis [3, 4]. Stress, depression and anxiety have been identified in some observational studies as potential factors that may modify and affect periodontal diseases [5, 6], but there are no similar data for peri-implantitis.

Changes in psychological conditions, the emergence of depression and stress factors can affect oral hygiene, smoking or alcohol consumption habits indirectly and can increase microbial dental plaque accumulation. Thus, individuals become more susceptible to unhealthy conditions. These factors may also alter the host immune response and can directly affect periodontal health [7].

Investigations have explained that psychological stress promotes the hypothalamus–pituitary–adrenal (HPA) axis activation, after which corticotrophin-releasing hormone (CRH) is secreted from the hypothalamus. Subsequently, adrenocorticotropic hormone (ACTH) release is stimulated from the pituitary gland and finally glucocorticoid secretions increase from the adrenal cortex [7, 8]. Glucocorticoids can inhibit immunoglobulin (Ig)A secretion, which can lead to a response in the immune system, cytokine expression and colonization of periodontal pathogens, and inhibit IgG secretion, which can allow pathogens to be recognized and phagocytized by neutrophils [7, 9, 10].

Glucocorticoid hormones exhibit their effects by binding to glucocorticoid receptors (GRs) [11]. GRs, members of the superfamily of nuclear receptors, bind glucocorticoids in cytoplasm and act as a transcription factor, inhibiting both CRH and ACTH secretion and synthesis. They play a critical role in the regulation of HPA axis feedback mechanisms and in stress adaptation [12, 13]. GRs have been identified in two isoforms: GRα, and GRβ. GRα binds glucocorticoids and mediates glucocorticoid effects but GRβ is unable to bind glucocorticoids [14, 15].

Another biological marker, which has been proposed to be sensitive to stress-associated changes, is salivary α-amylase (sAA). Stimulation of the autonomic nervous system (ANS) that controls the salivary glands results in release of sAA. It is known that sAA plays a role in the digestion of starch and has an inhibitory function against micro-organisms, but at the same time increases corresponding to the response of the sympathetic nervous system (SNS) to both psychological and physical stressors [16, 17].

Although the first step of peri-implant disease pathogenesis is related to micro-organisms, the immuno-inflammatory response with pro- and anti-inflammatory cytokines plays an important role in disease progression [18]. The pro- and anti-inflammatory cytokine balance determines the severity of inflammatory diseases [19], and this local host response to bacterial biofilm is in immunological and biochemical aspect, which is very similar in both periodontal and peri-implant diseases [20].

In clinical practice, peri-implant conditions are assessed through clinical indices such as probing depth, bleeding on probing, suppuration, and their combination with radiographic bone [21, 22]. Nevertheless, variables like probing orientation, the configuration of prosthetic structures, and tissue biotype can impact the outcomes of clinical assessments. At the same time, these clinical diagnostic tools provide information about the present inflammation status, but does not provide adequate prediction of the activity and severity of tissue destruction. To ensure diagnostic clarity, implantology calls for innovative diagnostic approaches such as the assessment of inflammatory biomarkers within biological fluids. Thus many studies evaluated the activity of systemic diseases, periodontal and peri-implant diseases or treatment efficacy with host derived proteins in saliva but to the best of our knowledge, no study has evaluated the psychological stress-related markers of peri-implantitis patients. Thus, the aim of this study was to determine the relationship between interleukin (IL)-1β, IL-6, IL-10, interferon (IFN)α inflammatory cytokines and the psychological stress-related markers, glucocorticoid receptor-α (GRα), and sAA gene expression levels in saliva samples obtained from healthy implants and peri-implantitis patients.

## Materials and methods

### Patient population

The study included a total of 50 systemically healthy subjects (16 females, 34 males aged 23 to 72 years) who were treated at Gazi University Department of Periodontology, Ankara, Turkey. The selected patients were informed regarding the details of the research and signed informed consent forms were received. This study was approved by the human subjects ethics board of Ankara University, Faculty of Dentistry (Protocol ID: 36290600/51) and was conducted in accordance with the Helsinki Declaration.

### Inclusion and exclusion criteria

This cross-sectional study included systemically healthy, partially edentulous subjects who had an implant-supported restoration functioning for at least two years. The cases were defined as peri-implantitis and healthy implants using the criteria in the consensus report of workgroup 4 of the 2017 World Workshop [23]. Peri-implantitis was defined as bleeding on probing (BOP) or suppuration, and increased pocket depth associated with radiographic bone loss. Healthy patients had no clinical signs of inflammation, BOP, suppuration, erythema or swelling.

Exclusion criteria were as follows for all groups: (1) non-surgical/ surgical periodontal or peri-implant therapy within the previous 6 months, (2) presence of periodontitis (i.e., suppuration and/or BOP in > 30% of the subgingival sites or any dental site with probing depth [PD] ≥ 4 mm), and localized (BOP ≥ 10% and ≤ 30%) or generalized (BOP > 30%) gingivitis (3) pregnancy (4) bruxism (5) antibiotics or anti-inflammatory medication usage within 6 months before the clinical examination, (6) regular intake of anticonvulsant, immunosuppressive, calcium channel blockers, antipsychotic/antidepressant drugs, (7) smoking.

### Clinical examinations

The same clinician recorded all the clinical examinations using a 0.5 mm Williams-type periodontal probe from the four sides of each implant (mesial, buccal, distal, lingual/palatal). The peri-implant clinical measurements were recorded as follows for each implant side: (1) pocket depth (PD), the distance between the gingival margin and the bottom of a peri-implant pocket (2) modified plaque index (mPI) [24], (3) modified gingival index (mGI) [24], (4) bleeding on probing (BOP).

### Questionnaire

The psychological stress level was evaluated with the hospital anxiety and depression scale (HAD) and state- trait anxiety inventory (STAI) questionnaire forms. The HAD scale is designed to measure the risk, level or severity of anxiety and depression in patients. The scale consists of two sub-scales, each with 7 items, assessing both anxiety (HAD-A) and depression (HAD-D) at the same time. The validity and reliability study of the questionnaire in Turkish was conducted by Aydemir [25]. The cut-off scores of the Turkish scale were calculated as 10/11 for the HAD-A sub-scale, and 7/8 for the HAD-D sub-scale. According to these results, patients with a score above the cut-off point are considered at risk for anxiety or depression.

The STAI questionnaire is composed of two sub-scales, each consisting of 20 items, which can measure state (STAI-I) and trait (STAI-II) of anxiety. Oner and Le Compte conducted the validity and reliability studies of the questionnaire in Turkish [26]. Scores can range from 20 to 80, higher scores indicates greater anxiety, and ≥ 40 indicates clinical symptoms of anxiety [27, 28]. Groups were divided into “a” or “b’’ according to the cut-off points of the stress level assessment scales (HAD-A ≥ 11, HAD-D ≥ 8, STAI-I ≥ 40, STAI-II ≥ 40).

The study participants were divided into four groups: patients with healthy implant and a score above the cut-off value of the stress level assessment scales (Group Ia, *n* = 16) or a score below the cut-off value of the stress level assessment scales (Group Ib, *n* = 9); patients with peri-implantitis and a score above the cut-off value of the stress level assessment scales (Group IIa, *n* = 15), and those with a score below the cut-off value of the stress level assessment scales (Group IIb, *n* = 10).

### Saliva sampling

All saliva samples were collected between 08.00 am -10.00 am to avoid circadian rhythm changes. Participants were instructed not to consume any nutrients or liquid for at least 1 h prior to sampling, and atraumatic brushing should be done 1 h before sampling. Then unstimulated whole saliva was collected into propylene collection tubes. If there was any blood or foreign substance in the collection tube, the sampling was repeated. Immediately after the appropriate collection of the saliva samples, they were stored in the RNAlater® (Sigma-Aldrich, Germany) to avoid RNA degradation. The samples were incubated at 4 °C for 24 h and then stored at -80 °C until RNA extraction.

### RNA extraction and reverse transcription

Saliva were put in a tube and subsequently, the IL-1β, IL-6, and IL-10, IFNα, GRα, sAA mRNA levels were evaluated. Gene expression levels of beta-actin (β-actin) were used as a reference. Total RNA was extracted from respective saliva samples using the TriPure isolation kit (Roche, Germany) under the manufacturer’s recommendation. RNA was suspended in diethylpyrocarbonate-treated water, DNAse-treated (Turbo DNA-free; Ambion Inc.), and stored at 70 °C until use. RNA concentrations were determined by the microvolume spectrophotometer (Nanodrop 1000; Nanodrop Technologies LLC, Wilmington, NC, USA). Afterward, 1 μg of total RNA was used to synthesize cDNA using the first-strand cDNA synthesis kit (Roche Diagnostics Co., Indianapolis, USA) as described by the manufacturer.

The housekeeping gene of β-actin was used as control by performing with both in-house PCR and qPCR methods. 2X SYBR Green dye with (10pmol/ μl) forward (F1) primer (0,5pmol/ μl) reverse (R1) primer (0,5pmol/μl), dionised water (4 μl) and cDNA (5 μl) were added and the final volume was adjusted to 20 μl.

### Real-time PCRs

The primers used to amplify mRNA corresponding to the IL-1β, IL-6, and IL-10, IFNα, GRα, sAA sequences for quantitative PCR analysis were designed using the Light Cycler probe design software (Roche Diagnostics GmbH, Mannheim, Germany). The primer sequences, the amplification profiles, and amplicon length are described in Table 1.

Quantitative PCR (qPCR) was carried out using the LightCycler System (Roche Diagnostics GmbH) as recommended by the manufacturer (FastStart DNA MasterPLUS SYBR Green; Roche Diagnostics Co., Indianapolis, IN, USA). Results were expressed as relative quantification to the β-actin gene expression levels.

**Table 1:** Primer sequences, amplification profiles and estimated amplicon length

Gene	Primer sequence(5’-3’)	Amplification profile	Amplicon size (bp)
		(temperature [°C]/ time [s])	
β actine	F: CCAACCGCGAGAAGATGA R:CCAGAGGCGTACAGGGATAG	95/10, 56/5, 72/8	97
IL-1β	**F: TACCTGTCCTGCGTGTTGAA** **R:TCTTTGGGTAATTTTTGGGATCT**	95/10, 56/5, 72/6	76
IL-6	**F:GATGAGTACAAAAGTCCTGATCCA** **R: CTGCAGCCACTGGTTCTGT**	95/10, 56/5, 72/6	130
IL-10	**F:TGCCTTCAGCAGAGTGAAGA** **R:GCAACCCAGGTAACCCTTAAA**	95/10, 56/5, 72/8	120
IFNα	**F:GCAGAAATCATGAGATCCCTCT** **R: TTGTTTTCATGTTGGACCAGA**	95/10, 56/5, 72/8	89
GRα	**F:CTGGGGGAATATCTGCTGAA** **R:TCCTAATTATGGTGAATTTCCTAGTTC**	95/10, 56/5, 72/6	113
sAA	**F: GTCTCTCCACCAAATGAAAA** **R: GGTATCTTTCCCACCAAG**	95/10, 56/5, 72/6	64

F: Forward, R: Reverse

qPCR stages were performed on LightCycler® Nano (Roche, Germany) with the denaturation (95^0^C, 600 s), cycling: 95^o^C 10 s 60^o^C 10 s., annealing 72^o^C 30 s for 40 cycles, then cooling with 40^o^C 600 s. The calculations were done according to the control gene expressions of the housekeeping gene of β-actin. Quantitations were performed by using LightCycler® Nano Software 1.1 and the data of the expressions of the chosen genes in the study were calculated according to the 2^(-∆∆Ct)^ method.

### Statistical analysis

Given the absence of similar studies in the literature, the sample size was determined based on the effect size. Assuming an effect size of 0.5 for the difference in IL-1β expression level changes among the groups (Ia-IIa, Ia-IIb, Ib-IIa, and Ib-IIb), a sample size calculation was performed using a One Way ANOVA test with a power of 0.80 and a significance level of 0.05. The analysis indicated that a minimum sample of 48 individuals would be sufficient for the study.

Analyses were performed using SPSS for Windows 11.5 software program. (SPSS Inc., Chicago, IL, USA). The compatibility of data with normal distribution was examined graphically and with the Kolmogorov-Smirnov test. Data are presented as mean ± standard deviation for normally distributed continuous variables, median (minimum-maximum) for non-normally distributed continuous variables and count, percentages for categorical variables. In the examination of a statistically significant difference between the categories of a qualitative variable with two categories in terms of a quantitative variable, the Student’s t-test was used if the normal distribution assumption was met; otherwise the Mann-Whitney U test was used. In the examination of a statistically significant difference between the categories of a qualitative variable with more than two categories in terms of a quantitative variable, the One Way ANOVA test was used if the normal distribution assumption was met, otherwise the Kruskal Wallis H test was used. The Chi-Square test was applied to compare the relationship between qualitative variables. *p* value < 0.05 was considered statistically significant.

## Results

Peri-implant clinical parameters were recorded and saliva samples were obtained from a total of 50 patients. The clinical variables are presented in Table 2, and all groups were well matched. Peri-implant clinical examinations datas and questionnaire scores of the study groups are presented in Table 3. In both the IIa and IIb groups, mGI, mPI, BOP and PD data were significantly higher compared with group Ia and group Ib (*p* < 0.001). There were no significant differences between groups Ia and Ib, or between group IIa and group IIb. In terms of the mean age and functional loading times of the implants, no significant difference was observed between all the compared groups.

**Table 2:** Description of the study population

variables	Group Ia (*n* = 16)		Group Ib (*n* = 9)	Group IIa (*n* = 15)	Group IIb (*n* = 10)	*p*
Age (years, mean ± SD)	53.06 ± 11.44		52.11 ± 16.70	56.00 ± 6.71	59.80 ± 7.24	0,359^a^
Gender: (F, M)	4 F, 12 M		4 F,5 M	5 F,10 M	3 F, 7 M	0,794^b^
Functional loading time of implants (years, mean ± SD)	5.56 ± 4.46		4.89 ± 2.85	5.33 ± 1.23	6.20 ± 1.81	0,333^c^
**Brushing frequency** **(n,%)**	< 2	7(43.75)	1(11.1)	5(33.3)	4(40.0)	O,398^b^
	≥ 2	9(51.25)	8(88.9)	10(66.7)	6(60.0)	

n: number of subjects, SD: standart deviation, F: Female, M: male

^a^: Student-t test, ^b^:Chi-square test, ^c^:Mann Whitney U test

**Table 3:** Clinical examinations of implants and questionnaire scores of the study population

Variables	Group Ia (*n* = 16)		Group Ib (*n* = 9)		Group IIa (*n* = 15)		Group IIb (*n* = 10)		
	mean ± SD	median (min-max)	mean ± SD	median (min-max)	mean ± SD	median (min-max)	mean ± SD	median (min-max)	*p*
PD (mm)	2.38 ± 0.63	2.33 (1.00–4.00)	2.67 ± 0.64	3.00 (1.25–3.10)	5.98 ± 0.45	6.00 (5.50–7.50)	5.97 ± 0.51	6.00 (5.50-7.00)	< 0.001^a^
mPI	0.66 ± 0.47	1.00 (0.00–1.00)	0.50 ± 0.61	0.00 (0.00-1.50)	1.15 ± 0.58	1.00 (0.30-2.00)	1.60 ± 0.52	2.00 (1.00–2.00)	0.001^a^
mGI	0.63 ± 0.50	1.00 (0.00–1.00)	0.56 ± 0.53	1.00 (0.00–1.00)	1.20 ± 0.56	1.00 (0.00–2.00)	1.60 ± 0.52	1.00 (1.00–2.00)	< 0.001^a^
BOP (%)	0.00	0.00 (0.00–0.00)	0.00	0 (0.00–0.00)	95.00 ± 10.3	100.00 (75.00-100.0)	97.50 ± 7.91	100.00 (75.00-100.00)	< 0.001^a^
HAD-A	**7.13 ± 3.74**	**7.00** **(1.00–17.00**	**3.67 ± 3.20**	**3.00** **(0.00–9.00)**	**8.27 ± 3.47**	**8.00** **(1.00–13.00)**	**2.20 ± 2.66**	**1.00** **(0.00–7.00)**	< 0.001^a^
HAD-D	**8.25 ± 3.64**	**7.50** **(4.00–18.00)**	**4.78 ± 2.44**	**5.00** **(1.00–7.00)**	**7.93 ± 1.98**	**8.00** **(5.00–13.00)**	**2.70 ± 2.45**	**1.50** **(0.00–7.00)**	< 0.001^a^
STAI-I	**47.75 ± 9.28**	**48.50** **(25.00–65.00)**	**30.22 ± 7.92**	**32.00** **(20.00–38.00)**	**46.27 ± 11.13**	**49.00** **(30.00–65.00)**	**28.60 ± 7.25**	**27.50** **(20.00–38.00)**	< 0.001^a^
STAI-II	**49.81 ± 9.21**	**44.50** **(41.00–73.00)**	**36.33 ± 4.47**	**37.00** **(28.00–39.00)**	**48.07 ± 7.63**	**48.00** **(30.00–55.00)**	**32.00 ± 5.06**	**31.00** **(26.00–38.00)**	< 0.001^a^

PD: pocket depth, mPI: modified plaque index, mGI: modified gingival index, BOP: bleeding on probing, HAD: hospital anxiety and depression scale, STAI: state- trait anxiety inventory scale, n: number of subjects, SD: standart deviation

^a^:Mann Whitney U test

There was a statistically significant difference among the groups Ia, Ib, IIa, IIb in terms of HAD-A variable (*p* < 0.001). After Bonferroni correction was applied to the subgroups that showed significance, the Mann Whitney U test was used. Binary groups with a significant difference were found to be Ia-IIb, IIa-IIb and Ib-IIa (*p* = 0.015, *p* = 0.001, *p* = 0.034). There was a statistically significant difference between the groups Ia, Ib, IIa, IIb in respect of the HAD-D variable (*p* < 0.001). Bonferroni correction was applied to the subgroups with significance and the Mann Whitney U test was used. Significant differences were determined between the groups Ia-IIb and IIa-IIb (*p* = 0.001, *p* < 0.001).

A statistically significant difference was found between groups Ia, Ib, IIa, IIb in terms of STAI-I (*p* < 0.001). The groups showing significance were found to be Ia-Ib, Ia-IIb, Ib-IIa and IIa-IIb when they were examined with the Tukey Post Hoc test (*p* < 0.001, *p* < 0.001, *p* = 0.001, *p* < 0.001). In respect of the STAI-II variable, there was a significant difference between all the groups (*p* < 0.001). The Mann Whitney U test was used after Bonferroni correction was applied to the subgroups with significance. Binary groups with significant results were found to be IIa-IIb, IIb-Ia, Ib-IIa and Ia-Ib (*p* < 0.001, *p* < 0.001, *p* = 0.008, *p* = 0.003).

**Fig. 1:**
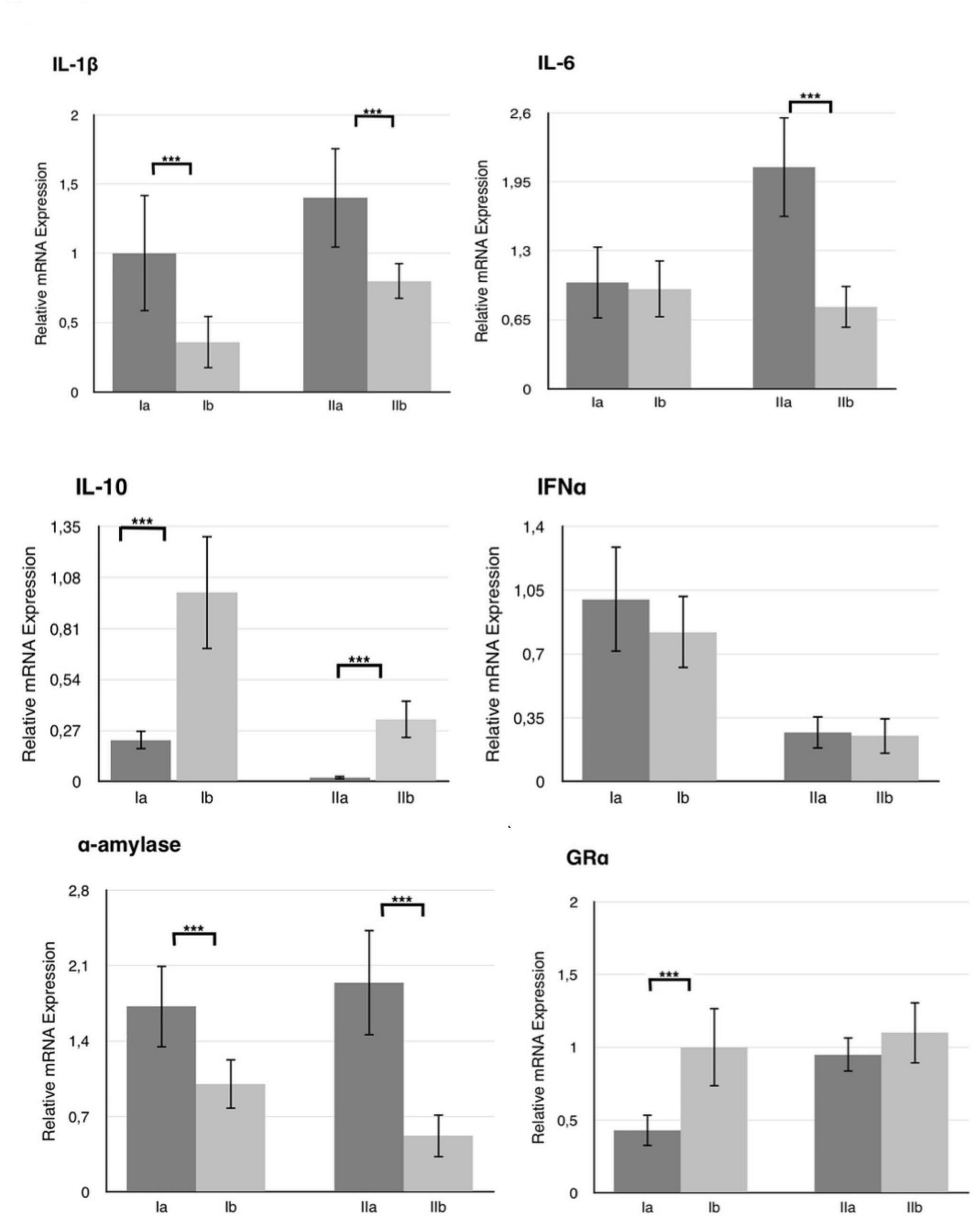
mRNA expression levels of IL-1β, IL-6, IL-10, IFNα, α-amylase, GRα in saliva samples of healthy and peri-implantitis patient groups. Expression levels were relative to housekeeping gene β-actine. (***) *p* < 0.001 represents the significant difference

The gene expression levels for each biological marker in all the groups are shown in Fig. 1. IL-1β and IL-6 expressions were significantly higher in group IIa compared with group IIb (*p* < 0.001). IFNα gene expression was similar in groups IIa and IIb. IL-10 gene expression was significantly lower in group IIa than in group IIb (*p* < 0.001). IL-1β gene expression was significantly higher in group Ia than in group Ib. IL-6 and IFNα gene expressions were similar in groups Ia and Ib. IL-10 gene expressions were significantly lower in group Ia compared to group Ib (*p* < 0.001).

In respect of sAA gene expressions in saliva samples, there was significantly higher expression in group IIa compared with group IIb (*p* < 0.001), and in group Ia compared to group Ib (*p* < 0.001). GRα gene expressions were significantly higher in group Ib than in group Ia (*p* < 0.001). GRα gene was expressed at lower levels in group IIa than in group IIb but the difference was not statistically significant (*p* = 0.065). When the participants were divided according to the stress level assessment scores, regardless of peri-implant health or disease status, group (Ia + IIa) was determined to have significantly lower GRα gene expression values than group (Ib + IIb).

## Discussion

In this study, the association between inflammatory cytokine levels and stress-related biomarkers was examined in order to evaluate the effect on peri-implantitis and peri-implant health of psychological stress, which is known to significantly affect quality of life and cause the development of many diseases.

The identification of biomarkers that play a role in the pathogenesis of peri-implantitis allows for a better understanding of the pathophysiological mechanism of the disease and allows evaluation of the immune status of the organism. Previous investigations have reported that pro-inflammatory cytokines and chemokines play a role in the early and advanced stages of peri-implant disease, leading to the process of inflammation and tissue destruction [19]. Schierano et al. showed that pro- and anti-inflammatory cytokines were released at varying levels at 4, 8, and 12 months after implant placement. This change in cytokine levels is explained by the attempts to stabilize the immune-inflammatory balance in peri-implant tissues after implant surgery [29]. In the current study, the implants of all participants had been functioning for at least 24 months and the mean duration of function was 5.58 years.

Saliva, known for its low cost, easy handling, usually available for sampling in large quantities and the presence of various biomarkers, including genomic material (mRNA, DNA) and proteins, offers notable advantages over other peri-implant fluids. The genomic material in its content is stable and in sufficient amounts for utilization in PCR procedures. In comparison to other fluids such as serum and peri-implant crevicular fluid, it offers an easy solution for storage, necessitates less technical precision and equipment during the collection process. Numerous studies use peri-implant sulcular fluid (PISF) as the preferred medium for assessing biomarkers in peri-implantitis patients [30, 31]. However, this methodology is technique-sensitive and demands a complicated toolkit. On the other hand, collecting saliva is a relatively straightforward technique that doesn’t necessitate extensive training when compared to obtaining PISF samples. Additionally, saliva sample collection is a fast and noninvasive method, which does not cause stress as blood collection may do [32]. Psychological stress-related studies have indicated that samples should be collected without activating any local reflex mechanisms [33]. In the light of this information, an unstimulated whole saliva collection protocol was applied in this study.

There are numerous studies evaluating the biomarkers that change due to peri-implant tissue inflammation in saliva samples [34–36]. Peri-implantitis has been linked to elevated salivary concentrations of IL1β [34, 35, 37]. IL6, and IL10 [35, 37], and these interleukins have been suggested as potential valuable indicators for the early detection and monitoring of this condition.

In the current study, HAD-A, HAD-D, STAI-I and STAI-II scales were used to determine the participants’ stress levels. These scales were used in many studies in which stress and anxiety were measured in dentistry. The validity and reliability of these questionnaires for Turkish population were tested before [25, 26]. These questionnaires, which the participants themselves have read and answered, eliminate the possibility that the researcher may affect or direct the participant [38].

The 2017 World Workshop on Periodontology report pointed out that stress is one of the environmental factors involved in periodontal breakdown [39]. There are studies showing that factors such as smoking, poor oral hygiene, diabetes, and genetic characteristics are risk factors for periodontal and peri-implant diseases and that psychological stress can also be a risk factor for periodontal disease [7, 40, 41]. However, to the best of our knowledge, there is no study in literature similar to the current research, which investigated the relationship between peri-implantitis and psychological stress.

Depression is one of the most common psychological disorders and studies have shown positive correlations with chronic inflammatory diseases such as autoimmune disease, inflammatory bowel diseases and allergies. This positive relationship is thought to be not only because of the depressive nature of chronic diseases, but also due to higher inflammatory cytokine expressions under these conditions [42]. According to recent opinions, depression does not develop due to a single neural network connection, but with multiple neural networks involving neurotransmitters, which function in the transmission of environmental stimuli to the brain, particularly stress [43]. Stressful life events affect and depress the immune system of subjects who then show depressive symptoms. Paik et al. investigated the effect of academic stress on the immune system and showed elevated levels of IL-1β, IL-6, and IL-10 [44]. These results are consistent with the current study results for IL-1β and IL-6 expression levels, although in our study, the IL-10 expression levels were lower in the groups with a high score in the stress level assessment scales. These IL-10 expression level values are consistent with the results of a study by Dhabhar et al. [45]. They evaluated pro- and anti-inflammatory cytokine concentrations in depressive patients and showed that IL-10 levels were statistically lower in depressive patients and IL-6 levels were higher but not at a statistically significant level in depressive patients. In healthy conditions, higher levels of IL-6 induce IL-10 expressions due to their anti-inflammatory and immune-regulatory effects but according to the study results, the inducing effects were seen at low levels in depressive patients [45]. In the current study, the groups with high scores in the stress level assessment scales, such as Ia and IIa, showed statistically lower IL-10 levels than groups Ib and IIb and IL-6 expression levels were statistically higher in group IIa than in group IIb.

Interferons are known as a large cytokine family that has antiviral, antitumor, anti-proliferative and immune-modulatory effects. IFNα, also known as type I interferon, is expressed by type 1 T helper cells and by fibroblasts against viral and bacterial stimuli. IFNgama(γ), known as type II interferon, has more powerful effects on immune system modulation and studies related to periodontal diseases have evaluated IFNγ much more than IFNα [46]. Recent studies have indicated that IFNα is a multifunctional cytokine, which causes anti-inflammatory cytokine activation and pro-inflammatory cytokine inhibition [47]. Wright et al. evaluated IFNα expressions in periodontitis patients and found elevated IFNα levels related with periodontal infection, suggesting that IFNα may play a role in periodontal disease pathogenesis [46]. The current study results demonstrated statistically significant higher levels of IFNα gene expression in group Ia than in group IIa, and in group Ib than in group IIb. Th1 cells are responsible for the cell-mediated immune response and producing IFNα, so the elevation in IFNα levels in the healthy groups of the current study may support the data that cell-mediated immune response is low level in peri-implantitis patients [48].

sAA is a notable protein of saliva that plays a role in the host response by acting as an inhibitory factor against micro-organisms. Sánchez et al. showed that the sympathetic system is activated by the inflammatory process in periodontitis and upregulates sAA to increase the salivary defence potential [48]. Consistent with the knowledge that sAA may be an indicator of the SNS, it has been also reported that sAA can be released in response to psychosocial stress. The results of the current study support this view as the sAA expressions were determined to be higher in groups Ia and IIa compared to groups Ib and IIb.

Previous studies have shown that inflammation and environmental factors such as stress affect GR functions in a negative way and cause glucocorticoid resistance in the organism [49, 50]. In an experimental animal study, GRα gene expressions were decreased with stress elevation and showed that stress may exacerbate the periodontitis progression mechanism. The current study results for GRα gene expression were similar, with significantly lower GRα gene expressions in group Ia than in group Ib, and although there was no statistically significant difference between groups IIa and IIb, lower GRα gene expression was observed in group IIa. When the participants were compared according to stress levels exclusively, group (Ia + IIa) showed significantly lower GRα gene expression values than group (Ib + IIb). Nevertheless, additional studies are necessary to confirm these findings, and determine whether GRα may be assessed in saliva as a stress-related marker in the same way as cortisol. To the best of the authors’ knowledge, the present study is the first study that assesses stress-related markers and inflammatory cytokines in saliva samples of peri-implantitis patients by using qPCR. Small sample size and lack of the analyzing of markers expression at protein level could be limitations of this cross-sectional study. Therefore, there is a need for further studies to correlate between the markers expression at protein levels with mRNA levels for better understanding of stress role in peri-implantitis pathogenesis.

## Conclusions

The aim of current study was to evaluate inflammatory cytokines and psychological stress with its related markers in peri-implantitis patients compared to individuals with healthy implants.

The findings obtained from this study indicate that stress may increase the inflammation associated with peri-implantitis, and in healthy individuals, stress alone is not sufficient to cause inflammation but may increase the susceptibility to inflammation by affecting cytokine expression levels. For the prevention of peri-implantitis or to reduce the prevalence, it could be useful to assess stress levels, identify individuals with stress and make these patients aware of their stress levels and even to take a multidisciplinary treatment approach guided by professional psychological support when necessary. However, there is a need for further investigations to be able to better understand the role of psychological stress in the pathogenesis of peri-implantitis.

## Data Availability

The data that support the findings of this study are available from the corresponding author upon reasonable request.
